# Pilot Study of Preconception Carrier Screening in Russia: Initial Findings and Challenges

**DOI:** 10.3390/genes17010003

**Published:** 2025-12-19

**Authors:** Andrei S. Glotov, Yulia A. Nasykhova, Tatyana E. Lazareva, Natalya M. Dvoynova, Elena S. Shabanova, Maria M. Danilova, Natalia S. Osinovskaya, Yury A. Barbitoff, Marianna A. Maretina, Elizaveta E. Gorodnicheva, Ziravard N. Tonyan, Anton V. Kiselev, Anastasiia A. Basipova, Olesya N. Bespalova, Igor Yu. Kogan

**Affiliations:** Department of Genomic Medicine, D.O. Ott Research Institute of Obstetrics, Gynaecology, and Reproductology, Mendeleevskaya Line 3, 199034 St. Petersburg, Russianatosinovskaya@mail.ru (N.S.O.); barbitoff@bk.ru (Y.A.B.); kumi-ori3000@yandex.ru (M.A.M.); elizavetagorodniceva2@gmail.com (E.E.G.); ziravard@yandex.ru (Z.N.T.); kiselev-anton-otta@yandex.ru (A.V.K.); anamikhajlova@gmail.com (A.A.B.);

**Keywords:** carrier screening, genetic variants, gene mutation, autosomal recessive disorders, X-linked disorders

## Abstract

**Background/Objectives**: This study reports on findings from the first preconception screening performed in Russia and provides a comprehensive discussion of the significant results and challenges faced during the implementation of the project. **Methods**: Using a targeted sequencing panel of 33 genes (associated with 29 autosomal recessive and 4 X-linked diseases), we analyzed 165 couples considering pregnancy. The screening design also included analysis of the frequent pathogenic variants in the *SMN1*, *DMD*, *CFTR*, and *CYP21A2* genes that may not be detected through the next-generation sequencing approach. The sequential screening protocol, wherein the female partner was tested first, was used. **Results**: The results revealed that 35.8% of women (*n* = 59) were carriers of at least one pathogenic or likely pathogenic (P/LP) variant, with 7.9% of women (*n* = 13) carrying variants in two or more genes. Notably, the analysis identified 5 deletions of exon 7 in the *SMN1* gene, 1 deletion of the *CYP21A2* gene, and 1 large duplication in the *DMD* gene in female participants. The most frequently identified pathogenic variants occurred in the *CYP21A2*, *GJB2*, *SERPINA1*, and *ATP7B* genes. The screening identified six couples (3.6% of the cohort) at high risk of having a child with an autosomal recessive or X-linked genetic disorder. **Conclusions**: This pilot study confirms the high clinical utility of the gene panel, effectively evaluating reproductive risk in couples without a known family history of monogenic diseases. The findings indicate that the observed frequencies of identified gene variants differ from those theoretically expected, with a notable percentage of identified couples being at relatively high risk. Furthermore, these results highlight the indispensable role of comprehensive genetic counseling both before and after testing to ensure an appropriate preconception testing algorithm and informed reproductive decision-making.

## 1. Introduction

To date, approximately 5000 known protein-coding genes are associated with single-gene disorders and traits [1]. According to the European Organization for Rare Disorders (EURORDIS), up to 30 million individuals in Europe are affected by rare diseases, with the majority (80%) of these conditions having a genetic origin [2]. Furthermore, it is estimated that 70% of rare diseases manifest in childhood, and many of these conditions are both disabling and life-threatening. Recent data indicate that autosomal recessive (AR) and X-linked recessive (XL) diseases affect approximately 30 out of every 10,000 live births [3].

Carrier screening (CS) is a genetic test used to assess an individual’s carrier status for pathogenic variants associated with recessive genetic conditions. It aims to identify individuals or couples who may be at risk of having a child with a monogenic disorder. CS allows prospective parents to learn about their reproductive risks before having an affected child, ideally before conception, and this knowledge obtained from CS enables informed decisions to be made regarding current or future pregnancies [4]. For couples in which both reproductive partners are heterozygous carriers of pathogenic (P) or likely pathogenic (LP) variants of the same gene, or if a woman is a carrier of such variants in an XL condition gene, reproductive choice encompasses several options. These include prenatal diagnosis (PD) or in vitro fertilization (IVF) with preimplantation genetic testing for monogenic disease (PGT-M), acceptance of the potential risks associated with having a child with a hereditary disease, donor gametes, the decision to abandon pregnancy, and adoption.

The initiatives of the reproductive CS for inherited diseases have been available since the 1970s. Traditionally, carrier screenings have been focused on heritable AR conditions with significant health impacts in childhood and high prevalence in certain ethnic groups that are geographically isolated or those with cultural norms and customs that limit random mating (Tay–Sachs disease for Ashkenazi Jewish population, haemoglobinopathies for individuals of Mediterranean, African, Middle Eastern and South East Asian ethnicity) [5]. In Israel, the national carrier screening program for reproductive purposes was launched in 2002. The screening algorithm considers the multicultural composition of the Israeli population and includes genes associated with diseases prevalent in isolated ethnic groups [6]. Another large-scale screening program is Dor Yesharim, which offers anonymous premarital screening to young single adults from Ultra-Orthodox Jewish communities. This program was established in 1983 and implemented around the world [6]. In recent years, it has become increasingly evident that carrier screening for specific conditions based on socially defined ethnic groups or self-identified origins can be inequitable and scientifically incorrect. This concern is especially important considering the rising proportion of the population with mixed ethnicity and the high rates of global migration. Considering the limitations of this testing strategy and the widespread availability and decreasing costs of next-generation sequencing technology, it is advisable and recommended to use an ethnically and population-neutral approach to carrier screening today [7]. The expanded approach of carrier screening provides testing for a broad range of recessive conditions regardless of ancestry but requires careful selection of candidate genes based on carrier frequency in the region, disease severity, and other factors [8]. Current guidelines from the American College of Medical Genetics and Genomics (ACMG) recommend screening for all individuals during pregnancy in preconception format. ACMG recommends the inclusion of genes with a carrier frequency of at least 1 in 200 that are associated with moderate to severe disease [7]. National expanded carrier screening programs or pilot projects are currently being implemented in China [9], Australia (Mackenzie’s mission) [8], the Netherlands [10], Romania [11] and several other countries.

Currently, in Russia, CS is not a routine practice and there are no national carrier screening programs in the country, but an increasing number of families are requesting it prior to pregnancy planning. Furthermore, despite the implementation of several population research projects using genomic technologies, there is a lack of genetic research in Russia that specifically addresses the preconception period. This demographic group is of particular interest, as it provides valuable insights into future population dynamics.

In this article, we present the results of the first preconception screening performed in Russia, as well as we discuss the significant findings and challenges that we encountered during the project implementation.

## 2. Materials and Methods

### 2.1. Recruitment

The preconception genetic carrier screening was performed as a pilot project supported by research funding (project number № 1022040700839-2-3.2.2). The objectives of the project were the development of an algorithm for expanded screening of common AR and XL diseases in the population of the Russian Federation and evaluation of its clinical utility. A total of 330 samples (165 couples) were enrolled in this study from 1 June 2024, to 31 October 2025.

Recruitment of couples for participation in the project was carried out by healthcare specialists (geneticists) during routine medical appointments. The information about the project, as well as an invitation to participate in it, was also announced as a part of educational and scientific events. All medical procedures, laboratory tests, and medical consultations performed within the framework of the project were offered to patients free of charge.

Inclusion criteria for all participants required that they were couples preparing for pregnancy, age between 18 and 40 years, any ethnicity, planning a natural pregnancy or the first IVF attempt. Individuals in a consanguine marriage were eligible to participate in the project.

Exclusion criteria applied to all participants were as follows: those who plan to use donor eggs or sperm for IVF; individuals with unknown or unclear causes of infertility; patients with an affected child or family history of genetic syndromes. Additional criteria for women: women with multiple unsuccessful attempts at IVF and with two or more miscarriages were not eligible. Additional criteria for men: men with azoospermia who did not have cryopreserved sperm samples were ineligible.

The study recruiting process included the following steps: submitting an online questionnaire followed by the review of data by a geneticist and remote medical counseling (if necessary). If applicants met the project criteria, they were sent an invitation to participate in the study and a detailed algorithm. Before donating blood, participants signed an informed consent form for participation.

The study was approved by the Institutional Review Board of the D.O. Ott Research Institute of Obstetrics Gynecology and Reproductology (St. Petersburg, Russia), No. 125 from 12 May 2023. Informed consent was signed by all prior to their inclusion in the study and to the processing of their personal and medical data. The study was performed in accordance with the Declaration of Helsinki.

### 2.2. Panel Design

The gene/disease selection in the panel applied in our project is mostly based on the recommendations provided by the ACMG organization. The gene panel included 33 genes associated with both AR and XL conditions, with a severe or moderate phenotype and a carrier frequency of ≥1/200. The panel also included genes associated with potentially common conditions in the region, such as DARS2-associated leukoencephalopathy, nephrotic syndrome type 1, and non-life-threatening conditions that can lead to disability, such as sensorineural hearing loss and Stargardt’s disease. Additionally, the panel included genes specific to certain ethnic groups living in the Russia, including Mediterranean fever. A complete list of the genes tested can be found in the Supplementary Table S1.

The screening algorithm included analysis of frequent pathogenic variants that may not be detectable through the exome sequencing method, including the deletion of exon 7 in the *SMN1* gene, large deletions or duplications in the *DMD* gene, the 21kb deletion in the *CFTR* gene, and 9 common variants in the *CYP21A2*.

### 2.3. Testing

**Figure 1:**
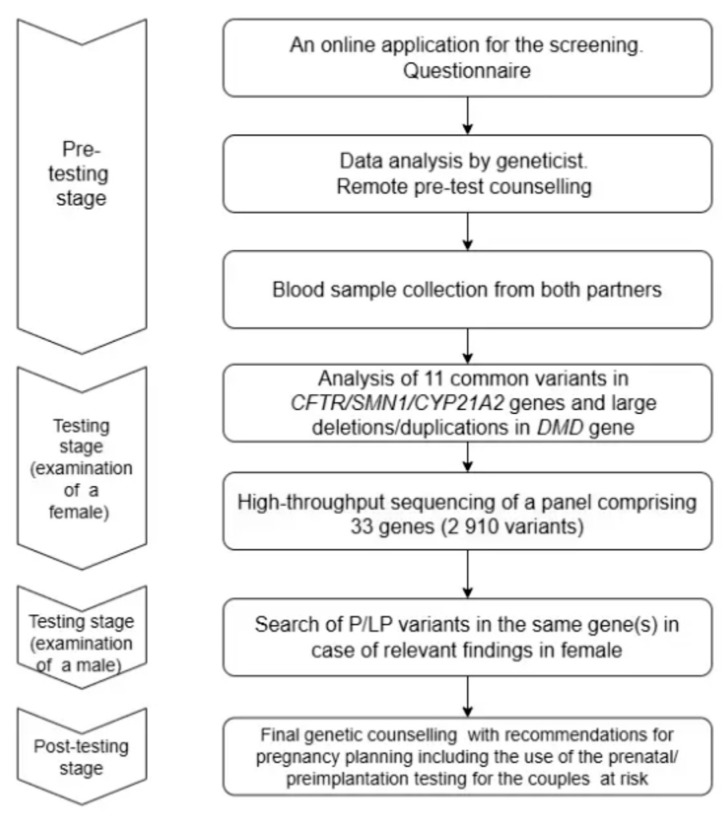
The flowchart of study design.

In this project we used a sequential screening protocol, in which a woman was tested first. The study of females was conducted according to an algorithm that included several stages of testing. A schematic overview of the experimental design is provided in Figure 1. At the initial stage, the analysis focused on large deletions and duplications in the *DMD* gene, as well as common pathogenic variants in the *CFTR*, *CYP21A2*, and *SMN1* genes. Subsequently, an NGS-based panel encompassing 33 genes was performed in female participants. If there were no P/LP variants detected at this stage, the couples received medical counseling and were provided with general recommendations for pregnancy planning, ensuring that the risk of monogenic conditions screened remained at a level not exceeding the population average. However, if at the preliminary stages of the survey a woman was found to have significant findings, we searched for corresponding variants in a male partner using appropriate technology (targeted sequencing, MLPA, RT-PCR, PCR). If both partners were identified as heterozygous carriers of P/LP variants in the same gene responsible for an AR condition, or in cases where a woman was a carrier of P/LP variants in the XL disease gene, then such couples were considered to have an increased risk of having a child with monogenic disease. These couples were offered genetic counseling with information about the possibility of disease prevention.

### 2.4. DNA Isolation

Peripheral blood samples from all participants were collected in 4 mL vials with EDTA. Genomic DNA was extracted from peripheral blood leukocytes using a protocol for salt/chloroform DNA extraction with modifications. The integrity of the DNA extracted was assessed using 3% agarose gel electrophoresis. The DNA purity was detected using a NanoDrop 2000 spectrophotometer (Thermo Fisher Scientific, Waltham, MA, USA). Finally, the DNA concentration was further measured by Qubit 2.0 Fluorometer (Invitrogen, Waltham, MA, USA).

### 2.5. Analysis of CYP21A2 Common Variants

Analysis of the most common variants in the *CYP21A2* gene, such as c.92C>T (p.Pro31Leu, rs9378251, P31L), c.293-13C>T, (rs6467, IVS2AS), c.332_339delGAGACTAC (p.Gly111Valfs, rs387906510, 8bp del), c.518T>A (p.Ile173Asn, rs6475, I173N), c.713T>A (p.Val238Glu, rs12530380, V238E), c.844G>T (p.Val282Leu, rs6471, V282L), c.955C>T (p.Gln319Ter, rs7755898, Q319X), c.1069C>T (p.Arg357Trp, rs7769409, R357W), c.1360C>T (p.Pro454Ser, rs6445, P454S), was performed using the PCR-RFLP method. To determine gene variants, we used two-step PCR with the initial amplification of *CYP21A2*. The primers were designed by means of Oligo 6.0 software and NCBI BLAST web tool v.2.17.0 (blast.ncbi.nlm.nih.gov, accessed on 17 December 2025). The sequences of the primers used and fragment size for each variant analyzed are given in Table S2, Supplementary Materials. The amplified fragments were separated in 7% polyacrylamide gel. *CYP21A2* gene deletions were determined by RT-PCR using fluorescently labeled primers and RT-PCR Thermocycler QuantStudio 5 (Thermo Fisher Scientific, Waltham, MA, USA) for sample amplification. The copy number of the *CYP21A2* gene was established relative performed relative to housekeeping gene (BGL) used as endogenous control in QuantStudio Design and Analysis Software v.1.5.1 (Thermo Fisher Scientific, Waltham, MA, USA). The sequences of the primers and fluorescently labeled molecular probes used are given in Table S3, Supplementary Materials.

### 2.6. Analysis of CFTR 21kb Deletion

The identification of the 21kb deletion (c.54-5940_273+10250del, CFTRdele2,3) in the *CFTR* gene was performed using duplex PCR with two pairs of primers [12]. The first pair flanked the boundaries of the deletion, while the second pair allowed for amplification of exon 10 of the CFTR gene, serving as a positive control. The sequences of the primers used are provided in Table S4 of the Supplementary Materials. The amplified fragments were separated using a 7% polyacrylamide gel.

### 2.7. Analysis of SMN1 Exon 7 Deletion

The quantitative analysis of SMN1 exon 7 deletions was performed using Genome-X SMA/TREC/KREC testing kit (Genome-Mix LLC, Saint-Petersburg, Russia) based on TaqMan RT-PCR technology. RT-PCR thermocycler QuantStudio 5 (Thermo Fisher Scientific) was used for sample amplification. The calculation of the SMN1 exon 7 copy number and deletion analysis was performed relative to housekeeping gene used as endogenous control in QuantStudio Design and Analysis Software v.1.5.1 (Thermo Fisher Scientific).

### 2.8. Analysis of DMD Exonic Deletions and Duplications

Detection of exonic deletions and duplications in the *DMD* gene was performed using multiplex ligation-dependent probe amplification (MLPA) with SALSA MLPA Probemixes P034-B2 DMD-1 & P035-B1 DMD-2 (MRC-Holland, Amsterdam, The Netherlands) according to the manufacturer’s protocol. Amplification products were analyzed by capillary electrophoresis on ABI Prism 3100 and 3130xl genetic analyzers (Thermo Fisher Scientific, Waltham, MA, USA). The original data were analyzed by Coffalyser.net software v.240129.1959 (MRC-Holland, Amsterdam, The Netherlands) according to the instructions.

### 2.9. Exome Libraries Preparation and Sequencing

Exome libraries were constructed using KAPA HyperPlus Kit (Roche, Basel, Switzerland), KAPA Unique Dual-Indexed Adapter Kit (Roche, Basel, Switzerland) for Illumina Platform, KAPA HyperCap Heredity Panel (Roche, Basel, Switzerland), KAPA HyperCapture Reagent Kit (Roche, Basel, Switzerland), KAPA HyperCapture Bead Kit (Roche, Basel, Switzerland), and MGIEasy Universal Library Conversion Kit (App-A) (MGI, Shenzhen, China) with standard input of 200 ng of gDNA as described in the manufacturers’ guidelines. The concentration of libraries was measured using Qubit dsDNA HS Assay Kit with Qubit 2.0 Fluorometer (Invitrogen, Waltham, MA, USA). Quality control of libraries was performed using the High Sensitivity DNA assay on the Agilent 2200 TapeStation (Agilent Technologies, Santa Clara, CA, USA). Barcoded libraries were pooled at equimolar ratios and paired-end sequenced with DNBSEQ-G50 platform (MGI, Shenzhen, China) according to the manufacturer’s instructions.

### 2.10. Targeted Amplicon Sequencing

For targeted sequencing analysis of the *SERPINA1, GJB2, ATP7B, GALT,* and *PKHD1* genes the amplicon-based enrichment AmpliSeq technology (Thermo Fisher Scientific, Waltham, MA, USA) was used. The custom panel containing the primers pairs to cover the exons of genes listed was designed using Ion AmpliSeq Designer online tool (Thermo Fisher Scientific). The libraries were constructed using Parseq Prep&Seq™ U-target DNA kit (Parseq Lab, Rotherham, UK) according to manufacturer’s instructions. Amplified samples were then sequenced on the DNBSEQ-G50 platform (MGI) according to the manufacturer’s instructions.

#### 2.10.1. Bioinformatics Analysis of Clinical Exomes Data

For the bioinformatic processing of the sequencing results from a clinical exome for preconception screening, an automated data analysis algorithm was developed. The pipeline was implemented on the Linux operating system using the Snakemake workflow management system’s abstract syntax, the Python v.3.1 programming language, and its associated libraries. The analysis pipeline involved the alignment of reads to the human reference genome (GRCh38) using the bwa-mem2 tool, accounting for ALT contigs (https://github.com/bwa-mem2/bwa-mem2, accessed on 11 November 2025). Alignment post-processing was performed using the software packages Samtools v.1.1.18 (https://www.htslib.org/, accessed on 11 November 2025) and the Genome Analysis ToolKit v.4.4.0.0 (GATK) (https://software.broadinstitute.org/gatk/, accessed on 11 November 2025). The protocol included the removal of PCR artifacts—specifically, read duplicates (using MarkDuplicates GATK)—to minimize library preparation and sequencing artifacts that could affect the reliability of variant calling. To assess the coverage of the target regions, quality metrics were calculated (using GATK CollectHsMetrics).

For variant identification, i.e., the detection of DNA sequence changes relative to the reference genome, the protocol utilized DeepVariant v.1.9, a tool based on a deep neural network (https://github.com/google/deepvariant, accessed on 11 November 2025). This was followed by joint genotyping of all samples in the run using GLNexus v.1.4.1 (https://github.com/dnanexus-rnd/GLnexus, accessed on 11 November 2025). The analysis of read groups from different samples allowed for removal of random errors from the study and enhanced sensitivity of the analysis by improving the detection of rare variants or variants in regions with low coverage in individual samples.

During the genetic variant annotation stage, the Ensembl Variant Effect Predictor v.110 (VEP) (https://www.ensembl.org/info/docs/tools/vep/index.html, accessed on 11 November 2025) was used. For each variant, the following information was added: an rsID, data on the variant’s effect and amino acid substitution, its frequency in general and Russian-specific populations (RUSeq) [13], its association with known hereditary diseases from public databases (gnomAD v.3.2.1., ClinVar, COSMIC, OMIM), and the results of pathogenicity prediction programs (SIFT, Polyphen, PROVEAN) based on data from the dbNSFP database (https://www.dbnsfp.org/about-us, accessed on 11 November 2025).

The final output consisted of two tables for each sample: the first was a list of annotated variants detected in the sample; the second contained details on the quality of the analysis performed (total number of reads, average read length, mean coverage, and the percentage of target regions with coverage of at least 10x).

#### 2.10.2. Variant Interpretation

The sequencing data was clinically analyzed for only pathogenic and likely pathogenic variants associated with carrier status for 33 autosomal-recessive and X-linked conditions. Although some variants were hypomorphic, they were included to minimize the couple’s risks. The annotated variants were visualized and ranked using proprietary score system. Variant classification followed the ACMG Standards and Guidelines for the Interpretation of Sequence Variants [14] with Sherloc refinement of the ACMG–AMP variant classification criteria [15] and Guidelines for the interpretation of massive parallel sequencing variants [16]. To assess the clinical relevance of the variants identified, dbSNP, ClinVar, OMIM, GeneBe, Franklin Genoox, VarSome, gene-specific databases (e.g., CFTR2), and literature data were used. Population frequency of the variants identified was indicated according to global allele frequency from the gnomAD v4.1.1 (Genome Aggregation Database).

#### 2.10.3. Statistics

All statistical tests were conducted using R v.4.3.1 (https://r-project.org/, accessed on 17 November 2025). Data visualization was performed by the following R packages: ggplot2 v.3.5.0 (https://ggplot2.tidyverse.org/, accessed on 17 November 2025), scales v.1.3.0 (https://scales.r-lib.org, accessed on 17 November 2025), and cowplot v.1.1.2 (https://wilkelab.org/cowplot/, accessed on 17 November 2025). For continuous variables, we used the unpaired Wilcoxon test. For categorical variables, a chi-squared test with the appropriate number of degrees of freedom was used. In all cases, a significance threshold of =0.05 was used for hypothesis testing.

## 3. Results

### 3.1. Description of the Group

**Figure 2:**
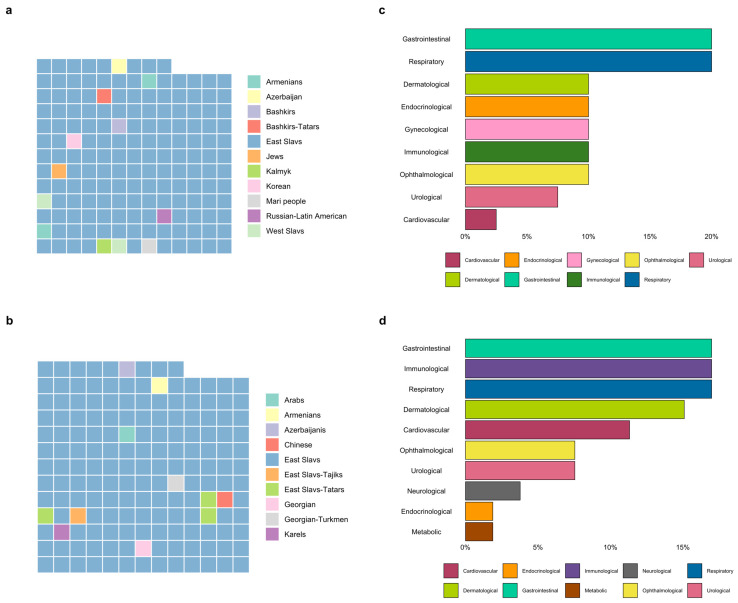
The majority of both female and male participants identified themselves as East Slavs (92.7% and 93.3%, respectively). The remaining participants belonged to a variety of other ethnic groups, the proportional distribution of which is displayed on (**a**) for females and (**b**) for males. Visual representation of chronic disease prevalence distribution among females (**c**) and males (**d**) in a studied group.

The study cohort comprised 165 couples (n = 330 participants), including one consanguineous. Women planning a pregnancy had a mean age of 29.9 ± 4.6 years, compared to 32.6 ± 9 years for men. East Slavs represented the majority ethnic group (93.3% of males, 92.7% of females), with the complete ethnic diversity profile illustrated in Figure 2. The presence of chronic disease was reported by 19.4% females (n = 32), with respiratory, gastrointestinal, representing the most common conditions. Among males, 28% reported chronic illnesses (n = 46), predominantly immunological, respiratory, gastrointestinal, dermatological, and cardiovascular disorders. The distribution of chronic disease burden is graphically illustrated through bar plots in Figure 2. Also, it should be noted that among couples who were planning the pregnancy, 30 couples had a family history of genetic inborn defects or infant death. Within the participant cohort, established genetic disorders were disclosed by 4.2% of females (n = 7) and 2.4% of males (n = 4). The constellation of hereditary conditions manifested in the study population included Gilbert’s syndrome, polycystic kidney and liver disease, and color blindness. In a cohort of 45 couples attempting pregnancy, 15 achieved a live birth. Of these 45 couples, 12 had conceived through ART (ovarian stimulation, artificial insemination, IVF, IVF+ICSI).

### 3.2. Assessment of the Carrier Frequency

Using the NGS gene set, we identified around 2910 of single nucleotide polymorphisms (SNP) in female patients, 58 variants were assessed as P/LP variants. In addition to NGS findings, 1 large duplication in *DMD*, 5 large deletions in *SMN1*, and 1 in *CYP21A2* have been revealed in the study cohort using supplementary techniques (see Section 2). Overall, 35.8% of female participants (n = 59) were carriers of at least one AR disease, including 10 individuals who were carriers of two AR diseases and four individuals who were carriers of three AR diseases (Table 1). The established frequency of AR and XL disease carriers is shown in Table 2.

We summarized the characteristics of the most commonly detected P/LP variants in our cohort in Table 3, with further information about all variants detected (including the ACMG classification criteria) available in Supplementary Table S5. Below, we will briefly describe the most notable of the findings.

Among the 33 genes examined, P/LP variants in the *CYP21A2* gene, associated with congenital adrenal hyperplasia (CAH), were most frequently detected in 165 females (7.9%). Variants c.844G>T (V288L) and c.955C>T (Q319X) were the most common: each was detected in 5 women. The overall carrier frequency for the *CYP21A2* gene aligns with our previous findings and global databases such as gnomAD v.4.1 [17]. The variants in *CYP21A2* are frequently absent from large-scale Russian genomic databases such as RUSeq because its highly homologous pseudogene, *CYP21A1P*, complicates accurate alignment and variant calling in short-read exome sequencing data, the primary source for these resources [13].

The *GJB2* (autosomal recessive deafness 1A) and *SERPINA1* (alpha-1-antitrypsin deficiency) genes harbored the second highest carrier frequency of P/LP variants in the study cohort (5.5%). The frequency of common variant c.35del (p.Gly12fs, rs80338939) (3%) in *GJB2* is aligned with population AF provided by RUSeq and gnomAD databases. The identified variants in the *SERPINA1* gene, c.1096G>A (p.Glu366Lys, rs28929474) and c.863A>T (p.Glu288Val, rs17580), were classified as LP. The frequency of the c.1096G>A variant was comparable to gnomAD v.4.1 frequency data, whereas frequency of c.863A>T variant was higher than established earlier in Northwest region of Russia and RUSeq (statistical difference insignificant), but lower than that in gnomAD dataset (chi-squared *p*-value = 0.02339, df = 1, X-squared = 5.1396).

Wilson disease, caused by pathogenic variants in the *ATP7B* gene, was the fourth most common autosomal recessive condition in our cohort, with a 3.6% carrier frequency. The variant c.3207C>A (p.His1069Gln, rs76151636), classified as LP, was the most frequent in our cohort. The observed frequency of it exceeded that reported in the Northwest Russian region, the Ruseq database, and gnomAD. Our finding that Wilson disease is among the most prevalent conditions in this cohort is consistent with our previous studies [13,18].

Pathogenic variants in the *CFTR* gene, associated with cystic fibrosis (CF), were the fifth most common, with a carrier frequency of 3%, while CF is one of the most common monogenic diseases in Russia, with well-established AF. However, the most common pathogenic variant, c.1521_1523delCTT (p.Phe508del, rs113993960), was detected in only 1 in 5 *CFTR* carriers in our cohort [19].Thus, the frequencies of F508del, CFTRdele2,3, and L138ins, which had been recommended for genetic monitoring in our region previously, were slightly lower than expected [20]. Specifically, the p.Phe508del allele frequency in our cohort was lower than that in the Northwest Russian population, the Ruseq database (statistical difference insignificant), and gnomAD (chi-squared *p*-value = 0.002465, X-squared = 9.1666, df = 1). This discrepancy may be attributed to the relatively small sample size of the present study.

Stargardt disease 1, caused by variants in the *ABCA4* gene, was the next most prevalent condition. This finding aligns with our previous report that it is among the most widespread monogenic diseases in the St. Petersburg region [18]. The *ABCA4* variant rs1800553 (c.5882G>A, p.Gly1961Glu) was the most frequent allele, identified in 60% (3/5) of carriers, and was classified as likely pathogenic.

We also identified five carriers of heterozygous deletions in exon 7 of the *SMN1;* the carrier frequency was 1 in 33. Interestingly, the frequency of heterozygous carriers, determined to be 1 in 47 in our previous large-scale screening of 36,140 newborns in St. Petersburg, was lower than in this study [21].

A carrier frequency of 2.4% was observed for Smith-Lemli-Opitz syndrome, caused by pathogenic variants in the DHCR7 gene. The nonsense variant c.452G>A (p.Trp151Ter, rs11555217) was identified, accounting for 75% (3/4) of the observed DHCR7 variants. The allele frequency of this variant in our cohort substantially exceeded that reported in the Ruseq (statistical difference insignificant) and gnomAD databases (chi-squared *p*-value = 1.147 × 10^−8^, X-squared = 32.574, df = 1).

Other autosomal recessive conditions were identified with an aggregate carrier frequency of 12.5%. These included disorders associated with pathogenic variants in the following genes: *DMD*, *ALPL*, *USH2A*, *ACADS*, *ACADM*, *BTD*, *PLOD1*, *PAH*, *IDUA*, *SLC26A2*, *GALT, PKHD1,* and *SLC26A4*. All variants are described in the Supplementary Table S5.

### 3.3. Couples at High Risk of AR or XL Disease

Our study revealed 5 couples in which both partners were heterozygous for pathogenic variants associated with the same AR condition. In one at-risk couple the female partner was identified to be a carrier of an XL disorder pathogenic variant. The compound heterozygous genotypes identified for the at-risk couples are shown in Table 4.

Thirteen female participants (7.9%) were identified as carriers of P/LP variants in more than one gene. Of these, nine women carried variants in two genes, and four women carried variants in three distinct genes. A notable case involved one female participant with a combination of three following variants: the duplication of exons 38-39 in the *DMD* gene (c.(5325+1_5326-1)_(5586+1_5587-1)dup), the variant rs1800552 (p.Gly1961Glu) in *ABCA4*, and the variant rs11555217 (p.Trp151Ter) in *DHCR7*. Her partner was shown to be a non-carrier of these AR diseases, so the couple received genetic counseling on pregnancy planning, taking into account the risk of transmitting the duplication in the *DMD* gene to the child.

Also, in one of the pairs examined, a pathogenic variant c.452G>A (p.Trp151*, rs11555217) in the *BTD* gene associated with biotinidase deficiency was found in a woman. A targeted study of this gene in her partner revealed a variant c.1270G>C (p.Asp424His, D444H). The enzyme activity in this variant homozygote is 50–60%, which does not cause a clinical phenotype. It is known that D444H in trans position with a P/LP variant in BTD gene is associated with partial biotinidase deficiency [22].Therefore, in this case patients do not need to prevent this monogenic disease. However, the results obtained about these variants in the *BTD* gene were provided to the reproductive couple, since in the case of a compound heterozygote, false positive results may be detected in their child during neonatal screening. Based on screening results, four at-risk couples in our study have resorted to or are actively considering assisted reproductive technologies (ART) with PGT-M, and one couple has prenatal diagnosis performed.

## 4. Discussion

The study reports the findings of the first pilot preconception carrier screening performed in Russia. The methodology employed in our project is predominantly aligned with the guidelines established by the ACMG and recommendations of the American College of Obstetricians and Gynecologists (ACOG) concerning disease/gene selection, as well as the testing design [23]. Additionally, in the development of the gene panel, we considered existing data on hereditary conditions prevalent in our region, particularly those associated with the various ethnic groups residing in the country.

When analyzing the study cohort, it was shown that the ethnic composition was largely representative of the St. Petersburg population, with approximately 93% being of East Slavic origin compared to the reported city average of 76.3% (rosstat.gov.ru, accessed on 17 November 2025). The average age of women in our cohort was 29–30 years, which aligns closely with the average maternal age predicted for St. Petersburg (31 years) [24].

As a result of the 33 genes studied, 35.8% of women (n = 59) were found to be carriers of P/LP variants in the genes analyzed. In 5 couples, both partners have P/LP variants in the same AR gene (*CFTR, GJB2, ATP7B, DHCR7*), and in one couple, a woman has a clinically significant variant in the DMD gene linked to Becker muscular dystrophy. Our data indicate that CAH, autosomal recessive deafness 1A, and alpha-1-antitrypsin deficiency are among the most frequent recessive conditions in this cohort. It is noteworthy that only autosomal recessive deafness 1A has previously been identified as one of the most common disorders in population studies in the region [18].

A significant finding of this study is the identification of a high frequency of pathogenic or likely pathogenic (P/LP) variants in the *CYP21A2* gene in our cohort. The high homology between *CYP21A2* and its pseudogene, *CYP21A1P*, often results in inaccuracies in the NGS data obtained from short reads. Consequently, a substantial proportion of pathogenic variants is frequently overlooked in standard NGS-based population studies and may be missed in the design of carrier screening panels that lack complementary, gene-specific validation methods. This explains why targeted studies often provide a more accurate characteristic of carrier frequencies. For instance, a recent project in China utilized capillary electrophoresis-based assays for study of the *CYP21A2* hotspot mutations, reporting a carrier rate of 1 in 66 [25]. Similarly, a nationwide screening study in Thailand employed an alternative method, identifying a nearly identical frequency of 1 in 65 [26]. In the Indian study, hotspot mutations in the *CYP21A2* gene were also analyzed in 1034 subjects using allele-specific genotyping based on PCR, and 101 carriers were identified (9.8%) [27]. Similarly, in a study involving 604 unrelated, unaffected Caucasian individuals of reproductive age from Western Romania, the *CYP21A2* gene (1:19) was identified as one of the genes in which pathogenic variants were the most common [11]. Our strategy, which integrates high-throughput sequencing with complementary molecular techniques, was specifically designed to address the limitations inherent in the NGS approach. It is crucial to emphasize that the clinical significance of precise screening for variants in the *CYP21A2* gene extends beyond the diagnosis of monogenic syndromes. Certain variants within this gene, particularly those associated with the non-classical form of CAH, may be linked to an increased risk of miscarriage (P31L, V282L, P454S). The presence of V282L variant in the heterozygous state of the *CYP21A2* gene has been previously shown to be associated with clinical manifestations related to increased androgen levels [28]. In our cohort study, the V282L variant was identified in 5 out of 13 individuals carrying variants in the *CYP21A2* gene. This finding underscores the vital role of *CYP21A2* gene screening in the context of reproductive medicine.

The carrier frequency of pathogenic variants in the *GJB2* gene, associated with autosomal recessive deafness 1A, observed in our cohort aligns with previously published data on the Russian population. It is noteworthy that Russia, similar to many other countries, exhibits a high prevalence of this condition. The potential for effective treatment has prompted increased attention from healthcare professionals toward neonatal screening for this disorder. Furthermore, the disabling nature of the disease and its high prevalence within the population underscore the necessity of including the *GJB2* gene in ECS panels.

The third gene with the highest score of P/LP variants in our study is *SERPINA1*. The high frequency of variants in this gene is unexpected and may be attributed to the characteristics of the population. Notably, we have identified LP variants in the *SERPINA1* gene. Previous research has demonstrated that even P/LP genotypes are not always linked to severe childhood liver disease, and the penetrance for adult-onset lung disease is incomplete, primarily influenced by environmental factors such as smoking. Consequently, while the S allele is considered pathogenic, its utility in a reproductive context is limited due to its lack of association with severe clinical manifestations. As a result, it is unlikely to influence decisions regarding reproductive interventions aimed at preventing the birth of a child with this genotype but it can be useful for planning the evaluations for future children based on surveillance recommendations for the disease [29].

Then, we evaluated the carrier rate for the next four most prevalent genes in our cohort: *ATP7B*, *ABCA4*, *SMN1*, and *CFTR*. The observed frequencies were largely consistent with the existing data from the Northwestern Russian population, and these genes are consistently reported among the top results in several national carrier screening programs [30,31,32,33,34,35]. This finding underscores the well-established principle that the distribution of recessive diseases varies significantly across ethnic groups. For example, cystic fibrosis, one of the most common life-limiting autosomal recessive conditions in Caucasian populations, is considerably less prevalent in Asian populations [34]. Conversely, β-thalassemia (which was included in our panel but for which no pathogenic variants were detected) is highly prevalent in Mediterranean, African, and Asian populations but is rare among individuals of Caucasian descent [35]. These findings emphasize the critical need to develop tailored screening programs and carrier data specific to a certain population, rather than relying on frequency data derived from other ethnic groups.

The fact that SMA and Wilson’s disease continue to be among the prevalent conditions in carrier screening, even despite the recent emergence of effective treatments for SMA, underscores the critical and ongoing importance of preventive genetic testing for these severe diseases. A significant difference from previous regional studies was the lower-than-expected frequency of *PAH* gene variants, associated with phenylketonuria, observed in our study. This discrepancy can be partially explained by the limited sample size, as well as the demographic characteristics of the Saint-Petersburg population, since it has been previously shown that the frequency of mutations in the *PAH* gene varies between different regions of the country [30].

One of the important parameters for evaluating the clinical utility of the screening algorithm is the analysis of the frequency of identified carriers for the genes included in the panel. In a Vietnamese carrier study, analysis of 540 genes in 338 Vietnamese women revealed that variants associated with AR or XL diseases were identified in 63.6% of the patients [36]. Similarly, Chan and colleagues conducted a study within the Chinese population utilizing an expanded carrier screening panel that encompassed 104 AR and XL conditions. This study involved a group of 123 pregnant women and 20 male partners, reporting that 58.7% (n = 84) of participants were carriers for at least one recessive condition [37]. In another Chinese study of expanded screening, including 220 diseases in 3024 individuals from southern and southwestern China, the carrier frequency of P/LP variants was 62.3% [30]. Recently, Chetruengchai and colleagues analyzed 114 recessive genes in 1642 individuals in Thailand and identified the carrier frequency for at least one AR disorder as 39% [38]. In our study, the analysis of 33 genes revealed a carrier frequency of 35.8%, which is lower than the frequencies reported in the aforementioned studies. However, when the panel was expanded to include 126 genes according to Schmitz and colleagues [37], the carrier frequency increased up to 46%, aligning with the ranges indicated in the previous literature. In our study, the analysis of 33 genes revealed a carrier frequency of 35.8%, which is lower than the frequencies reported in the aforementioned studies. However, when the panel was expanded to 126 genes, according to Schmitz and colleagues, the carrier frequency increased up to 46%, aligning with the ranges indicated in the literature. It confirms that the expansion of the NGS panel of genes for analysis is a promising area of research for our cohort aimed at enhancing the detection rate of P/LP variants.

In evaluating the outcomes of the screening algorithm, it is also crucial to assess the identification rate of couples at risk of having a child with a genetic disorder. This rate varies across different studies, ranging from 1.7% to 9.5% [30,31,32,33]. Specifically, the results from the Mackenzie’s Mission project in Australia indicated that among 10,038 couples screened, 175 (1.9%) were identified as having an increased probability of conceiving a child with a genetic disease [33]. In the context of extended carrier screening conducted in Israel, 2.6% of couples at risk were identified, with both partners being carriers of the same AR disease or with the female partner being a carrier of an XL disorder. Similarly, the study by Strauss and colleagues in New York reported that 71.85% of participants were identified as carriers, with an at-risk couple percentage of 9.46% [39]. It is noteworthy that in our study, 35.8% of the participants were identified as carriers of at least one AR or XL disease, with 3.6% of couples classified as at risk. These findings are consistent with previously published data, despite the limited panel of genes and cohort examined.

This finding suggests that the approach employed, which utilizes a relatively small gene panel for screening, is feasible. In contrast, most of the previously implemented projects relied on ECS panels. It is important to note that the composition of gene panels varies significantly across studies, often influenced by financial resources and the existing knowledge of population frequencies of diseases and genetic variants.

The number of genes and genetic variants included in the carrier screening panel is currently being discussed. A recent study analyzed over 700,000 exomes from gnomAD v.4.1.0, encompassing eight distinct ancestries, to estimate the carrier frequency of P/LP variants in 2987 genes associated with autosomal recessive (AR) conditions. Following an expert curation based on clinical severity, a total of 286 genes were identified as meeting the criteria for inclusion in carrier screening. Simulations indicated that pan-ethnic screening panels provide advantages for individuals of diverse or admixed ancestry, whereas ancestry-specific panels may be more suitable for genetically homogeneous populations [37]. Notably, only six genes (2.1%) exhibited a carrier frequency greater than or equal to 1 in 200 across all populations: *ABCA4, CYP21A2, FLG, GJB2, KCNE1,* and *PAH*. It is important to highlight that three of these genes are included in the panel utilized for this study.

Numerous studies have focused on the analysis of genetic variants associated with the risk of reproductive disorders [40]. Notably, research conducted by Guo et al. demonstrated that the highest genetic burden was observed in couples seeking medically assisted reproduction who had a history of fetal loss as well as second- or third-trimester abnormalities and postnatal complications [40].

In our comparison of the data with the PLOV database, we identified only one genetic variant (present in two patients) associated with early termination of pregnancy, specifically a homozygous mutation in the fetus (c.3207C>A in the *ATP7B* gene) [41]. Among the partners of these women, none of the men were found to have variants in the same gene. Additionally, it is noteworthy that the presence of the c.293-13C>T (rs6467, IVS2AS) variant in the *CYP21A2* gene in 5 out of 13 female carriers may also contribute to early pregnancy termination due to maternal factors [42]. This information holds significant implications for couples, as even in the absence of a risk for having an affected child, a planned pregnancy may still not occur or may be terminated. In the recently published results of the WES-based neonatal screening in the Russian population, the frequency of several diseases was found to differ significantly from that reported in the present study and previously published databases [43]. These discrepancies are likely attributable to limitations caused by the design of neonatal screening programs. Specifically, the exclusive use of the NGS approach along with the late onset of certain monogenic disorders and associated infant mortality rates may substantially distort the true population dynamics. It is therefore highly inadvisable to use the results of neonatal screening to calculate carrier frequencies for reproductive planning, as they may misrepresent the actual allele frequencies among individuals of reproductive age.

It also is crucial to highlight the significant role of genetic counseling for couples undergoing preconception screening. It encompasses the analysis of family history and pedigree to determine the appropriate scope of testing (pre-test counseling) and culminates in comprehensive counseling for the interpretation of genetic results and assistance in making informed reproductive decisions if risk is identified. Genetic counseling is a valuable tool to help individuals understand and adapt to their test results.

Our research has several limitations. One of them is the size of the analyzed sample. However, the identified carrier rate and the proportion of couples at risk are comparable to those observed in other populations, thereby supporting the representativeness of our findings despite the small sample size. Also, in our study, the partners were tested sequentially, with the female partner undergoing testing first; the male partner was subsequently tested only for the genes in which P/LP variants were identified in the female partner. Among the limitations of this study was that men were tested only for their couple’s risk profile. This approach carries the risk of missing P/LP variants in genes that do not correspond to those of the female partner. Additionally, a notable limitation of this method is the extended duration of the study compared to the simultaneous testing of both partners. However, this approach encourages the utilization of simpler and cost-effective laboratory testing methods, thereby making screening more accessible for implementation in a mass screening strategy.

In continuation of this pilot study, the findings obtained in this study will be employed to inform the design and implementation of a larger-scale project. Furthermore, we aim to broaden the scope of our analysis by increasing the number of genes assessed through NGS technology. This expanded genetic analysis will facilitate a more comprehensive grasp of the genetic factors contributing to reproductive outcomes. Additionally, we plan to closely monitor reproductive attempts of couples identified as high-risk ones, which enables us to evaluate the clinical implications of our findings and potentially develop targeted interventions. The integration of these approaches will enhance our ability to identify at-risk populations and contribute to the advancement of personalized reproductive healthcare.

In this study, we demonstrated that preconception carrier screening is a feasible approach for identifying couples at risk of having a child with a monogenic syndrome. A critical aspect of this process is the careful selection of the study’s scope and design, along with the determination of the gene panel, taking into account the ethnic and regional characteristics of the population, which are essential to ensure the effectiveness of the screening program.

## 5. Conclusions

This pilot study reports the findings of the first preconception carrier screening conducted in Russia. The comprehensive strategy employed, which integrated high-throughput sequencing with complementary molecular methods, proved highly effective. It successfully identified variant carriers and at-risk couples with a yield comparable to that of large-scale, expanded carrier screening studies. The most frequent P/LP variants were found in the *CYP21A2*, *GJB2*, *SERPINA1*, *CFTR*, *ABCA4*, *SMN1* and *ATP7B* genes among the participating women. Critically, the screening identified six couples (3.6% of the cohort) at high risk of having a child with a severe autosomal recessive or X-linked genetic disorder, confirming the high clinical utility of the gene panel for assessing reproductive risk in couples without a known family history of monogenic diseases. Although the sample size of this research is limited, it is nonetheless sufficient to draw meaningful conclusions about the program’s effectiveness and to formulate the foundational principles for organizing mass-scale preconception screening in Russia. The data provides a crucial proof-of-concept, demonstrating that such an initiative is both technically feasible and clinically valuable within the Russian healthcare context. The high-risk couple detection rate aligns with global data, underscoring the significant, previously unquantified burden of monogenic diseases that could be mitigated through proactive screening.

## Figures and Tables

**Table 1:** List of individuals who are carriers of 2 and more AR diseases.

ID	Gene	Variant
1	*CFTR*	rs113993960 (c.1521_1523del)
	*GJB2*	rs80338939 (c.35del)
13	*SMN1*	del7
	*DHCR7*	rs11555217 (c.452G>A)
18	*CFTR*	rs113993960 (c.1521_1523del)
	*SLC26A2*	c.310del
21	*CYP21A2*	rs7755898 (c.955C>T)
	*CFTR*	rs75961395 (c.254G>A)
65	*GJB2*	rs80338939 (c.35del)
	*SERPINA1*	rs28929474 (c.1096G>A)
80	*ATP7B*	rs76151636 (c.3207C>A)
	*CYP21A2*	rs7755898 (c.955C>T)
83	*PAH*	rs5030858 (c.1222C>T)
	*SERPINA1*	rs28931570 (c.187C>T)
90	*DHCR7*	rs11555217 (c.452G>A)
	*GJB2*	rs80338939 (c.35del)
123	*CYP21A2*	rs7755898 (c.955C>T)
	*DHCR7*	rs749076525 (c.651C>A)
158	*SMN1*	del7
	*SERPINA1*	rs17580
11	*DMD*	dup 38-39 ex c.(5325+1_5326-1)_(5586+1_5587-1)dup
	*ABCA4*	rs1800552 (c.5693G>A)
	*DHCR7*	rs11555217 (c.452G>A)
85	*ATP7B*	rs191312027 (c.2605G>T)
	*CFTR*	rs115545701 (c.220C>T)
	*SMN1*	del7
102	*ACADM*	rs147559466 (c.127G>A)
	*ATP7B*	rs76151636 (c.3207C>A)
	*CYP21A2*	rs12530380/rs7755898 (c.710T>A/c.955C>T)
140	*ABCA4*	rs61749420 (c.1957C>T)
	*CYP21A2*	rs6471 (c.844G>T)
	*PAH*	rs5030858 (c.1222C>T)

**Table 2:** Frequency of disease carrier in the study cohort.

Gene	Phenotype (MIM Number)	Number of Cases Identified	Carrier Frequency (1 in N)
*CYP21A2*	Adrenal Hyperplasia, Congenital, Due to 21-Hydroxylase Deficiency (201910) Hyperandrogenism, nonclassic type, due to 21-hydroxylase deficiency included	13	1 in 13
*GJB2*	Sensorineural nonsyn-dromic hearing loss (604418)	9	1 in 18
*SERPINA1*	Alpha-1-Antitrypsin Deficiency (613490)	9	1 in 18
*ATP7B*	Wilson Disease (277900)	6	1 in 28
*CFTR*	Cystic fibrosis (219700); Congenital bilateral absence of vas deferens (277180)	5	1 in 33
*ABCA4*	Stargardt Disease 1 (248200); Cone-rod dystrophy 3 (604116); Retinitis pigmentosa 19 (601718)	5	1 in 33
*SMN1*	Spinal Muscular Atrophy-1, 2, 3, 4 (253300, 253550, 253400, 271150)	5	1 in 33
*DHCR7*	Smith-Lemli-Opitz Syndrome (270400)	4	1 in 41
*GALT*	Galactosemia (230400)	3	1 in 55
*PKHD1*	Polycystic kidney disease 4, with or without hepatic disease (263200)	3	1 in 55
*SLC26A4*	Deafness, autosomal recessive 4, with enlarged vestibular aqueduct (600791); Pendred syndrome (274600)	2	1 in 83
*PAH*	Phenylketonuria (261600)	2	1 in 83
*IDUA*	Mucopolysaccharidosis Ih (607014); Mucopolysaccharidosis Ih/s (607015); Mucopolysaccharidosis Is (607016)	2	1 in 83
*DMD*	Becker muscular dystrophy (300376); Duchenne muscular dystrophy (310200)	1	1 in 165
*ALPL*	Hypophosphatasia (146300, 241510, 241500)	1	1 in 165
*USH2A*	Retinitis pigmentosa 39 (613809); Usher syndrome, type 2A (276901)	1	1 in 165
*ACADS*	Acyl-Coa Dehydrogenase, Short-Chain, Deficiency of (201470)	1	1 in 165
*ACADM*	Acyl-CoA dehydrogenase, medium chain, deficiency of (201450)	1	1 in 165
*BTD*	Biotinidase deficiency (253260)	1	1 in 165
*PLOD1*	Ehlers-Danlos syndrome, kyphoscoliotic type, 1 (225400)	1	1 in 165
*SLC26A2*	Achondrogenesis Ib (600972); Atelosteogenesis, type II (256050); Diastrophic dysplasia (222600); Epiphyseal dysplasia, multiple, 4 (226900)	1	1 in 165

**Table 3:** The most important findings in the study cohort.

Gene	Nucleotide Change	Protein Change	dbSNP	Cohort AF	gnomAD_ allele Frequency v4.1.0	Northwest Russia, AF	Ruseq AF (Healthy)
*CYP21A2*	c.844G>T	p.Val282Leu	rs6471	0.0303	0.005	-	-
*CYP21A2*	c.955C>T	p.Gln319Ter	rs7755898	0.0303	0.0008961	0.00000	-
*GJB2*	c.35del	p.Gly12fs	rs80338939	0.0303	0.00705	0.01837	0.01521
*SMN1*	deletion of the 7 ex			0.0303	-	-	-
*SERPINA1*	c.863A>T	p.Glu288Val	rs17580	0.02424	0.03636	0.00669	0.007113
*SERPINA1*	c.1096G>A	p.Glu366Lys	rs28929474	0.01818	0.01586	-	-
*ABCA4*	c.5882G>A	p.Gly1961Glu	rs1800553	0.01818	0.003406	0.00746	0.009775
*DHCR7*	c.452G>A	p.Trp151Ter	rs11555217	0.01818	0.0007107	0.00658	0.005045
*ATP7B*	c.3207C>A	p.His1069Gln	rs76151636	0.01212	0.0009435	0.00618	0.005651
*CFTR*	c.1521_1523del	p.Phe508del	rs113993960	0.00606	0.01193	0.00623	0.008021

**Table 4:** The P/LP variants identified in the same gene among couples.

Family ID	Gene	Female Partner P/LP Variant	Male Partner P/LP Variant
22	*ATP7B*	c.3688A>G (p.Ile1230Val, rs200911496)	c.3207C>A (p.His1069Gln, rs76151636)
66	*CFTR*	c.1397C>G (p.Ser466Ter, rs121908805)	c.1521_1523del (p.Phe508del, rs113993960)
90	*DHCR7*	c.452G>A (p.Trp151Ter, rs11555217)	c.964-1G>T (rs138659167)
41	*GJB2*	c.35del (p.Gly12fs, rs80338939)	c.101T>C (p.Met34Thr, rs35887622)
65	*GJB2*	c.35del (p.Gly12fs, rs80338939)	c.109G>A (p.Val37Ile, rs72474224)

## Data Availability

The original contributions presented in this study are included in the article/Supplementary Materials. Further inquiries can be directed to the corresponding author(s).

## Supplementary Materials

The following supporting information can be downloaded at: https://www.mdpi.com/article/10.3390/genes17010003/s1, Table S1: Genes and associated disorders covered by the screening panel; Table S2: Primer sequences of the variants in *CYP21A2* gene; Table S3: Sequences of the real-time PCR primers and the fluorescently labeled molecular probes; Table S4: Primer sequences of the variants in *CFTR* gene; Table S5: The list of identified P/LP variants in the gene screening panel.

## Abbreviations

The following abbreviations are used in this manuscript:

ACMG	American College of Medical Genetics and Genomics
AR	Autosomal Recessive
ART	Assisted reproductive technologies
CS	Carrier screening
IVF	In Vitro Fertilization
P/LP	Pathogenic or Likely Pathogenic variant
PD	Prenatal diagnosis
PGT-M	Preimplantation genetic testing for monogenic disease
