# Supplementary material for: Pilot Study of Preconception Carrier Screening in Russia: Initial Findings and Challenges
Source: Genes (Basel). 2025 Dec 19;17(1):3. doi: 10.3390/genes17010003 (PMC12841009; doi:10.3390/genes17010003)
Supplement: Supplementary file 1 [file genes-17-00003-s001.zip › genes-4033716-supplementary.pdf]

Pilot Study of Preconception Carrier Screening in Russia: Initial Findings and Challenges

Andrei S. Glotov [1], Yulia A. Nasykhova[1], Tatyana E. Lazareva[1], Natalya M. Dvoynova[1], Elena S. Shabanova[1], Maria M. Danilova[1], Natalia S. Osinovskaya[1], Yury A. Barbitoff[1], Marianna A. Maretina[1], Elizaveta E. Gorodnicheva[1], Ziravard N. Tonyan[1], Anton V. Kiselev[1], Anastasiia A. Basipova[1], Olesya N. Bespalova[1], Igor Yu. Kogan[1]

1- Department of Genomic Medicine, D.O. Ott Research Institute of Obstetrics, Gynaecology, and Reproductology, Mendeleevskaya Line 3, 199034 St. Petersburg, Russia

Supplementary Table S1. Genes and associated disorders covered by the screening panel.

№	Disease name	Gene	Gene OMIM	Inheritance pattern
1	Cystic fibrosis	*CFTR*	602421	AR
2	Spinal muscular atrophy	*SMN1*	600354	AR
3	Phenylketonuria	*PAH*	612349	AR
4	Sensorineural nonsyndromic hearing loss	*GJB2*|*GJB6*	121011/604418	AR
5	Pendred syndrome	*SLC26A4*	605646	AR
6	Smith-Lemli-Opitz syndrome	*DHCR7*	602858	AR
7	Wilson-Konovalov syndrome	*ATP7B*	606882	AR
8	Stargardt disease/retinitis pigmentosa	*ABCA4*	601691	AR
9	Leukoencephalopathy with predominant brainstem and spinal cord involvement and elevated lactate (LBSL)	*DARS2*	610956	AR
10	Polycystic kidney and/or liver disease, type 4	*PKHD1 (FCYT)*	606702	AR
11	Ehlers-Danlos syndrome, kyphoscoliotic type	*PLOD1*	153454	AR
12	Meckel syndrome type 1/Bardet-Biedl syndrome type 13/Joubert syndrome type 28	*MKS1*	609883	AR
13	Hemophilia A	*F8*	300841	XLR
14	Gaucher disease	*GBA*	606463	AR
15	Biotinidase deficiency	*BTD*	609019	AR
16	Deficiency Ornithine transcarbamylase	*OTC*	300461	XLR
17	Duchenne-Becker muscular dystrophy	*DMD*	300377	XLR
18	Antitrypsin deficiency	*SERPINA1 (AAT)*	107400	AR
19	Hypophosphatasia	*ALPL*	171760	AR
20	Nephrotic syndrome type 1 (Finnish type)	*NPHS1*	602716	AR

21	Methylmalonic aciduria and homocystinuria	*ACSF3*	614245	AR
22	Methylmalonic aciduria and homocystinuria, B12-dependent	*MMACHC*	609831	AR
23	Galactosemia	*GALT*	606999	AR
24	Congenital adrenal hyperplasia	*CYP21A2*	613815	AR
25	Homocystinuria	*CBS*	613381	AR
26	Usher syndrome type 2A	*USH2A*	608400	AR
27	Short-chain acyl-CoA dehydrogenase deficiency	*ACADS*	606885	AR
28	Medium-chain acyl-CoA dehydrogenase deficiency	*ACADM*	607008	AR
29	Long-chain acyl-CoA dehydrogenase deficiency	*ACADVL*	609575	AR
30	Mucopolysaccharidosis II (Hunter syndrome)	*IDS*	300823	XLR
31	Mucopolysaccharidosis I (Hunter syndrome) Hurler-Scheie)	*IDUA*	252800	AR
32	Familial Mediterranean fever	*MEFV*	608107	AR, AD
33	Diastrophic dysplasia/achondrogenesis type Ib	*SLC26A2*	606718	AR

Supplementary Table S2. Primer sequences of the variants in *CYP21A2* gene

Variant name	Primer sequence (5'-3')	Fragment size (bp)
Gene-specific primers	F:GCTTCTTGATGGGTGATCAATT<br>R:TCTCGCACCCCAGTATGACT	3127
P31L	F:CTACACAGCAGGAGGGATGGC<br>R:AGCAAGTGCAAGAAGCCCGGGGCAAGCTG	195
IVS2AS	F:TTCATCAGTTCCCACCCTCCAGCCCCGA<br>R:CTTCTTGTGGGCTTTCCAGA GCAGGTA	115
I173N	F:GAGGAATTCTCTCTCCTCACCTGCAGCATTA<br>R:TTGTCGTCCTGCCAGAAAAGGA	159
V238E	F:AGCAGGCCATAGAGAAGAGGGATCACATC<br>R:TGCAAAAGAACCCGCCTCATAG	144
V282L	F:TGCAGGAGAGCCTCGTGGCAGG<br>R:ACGCACCTCAGGGTGGTGAAG	212
Q319X, R357W	F:GCTGGGGCAGGACTCCACCCGA<br>R:GTGCGGTGGGGCAAGGCTAAGGGCACAACTG	196

P454S	F:TCCCCGCTGCCGCTGAACGC<br>R: GGGCAGGGCGTCCCCGGAGT	208

Supplementary Table S3. Sequences of the real-time PCR primers and the fluorescently labeled molecular probes

Gene/probe	Primer sequence (5′→3′)
CYP21A2	F:ACCTGTCCTTGGGAGACTAC<br>R:TTACCTCACAGAACTCCTGGGT
CYP21A1P (pseudogene)	F:GGACCTGTCGTTGGTCTCT<br>R:TTACCTCACAGAACTCCTGGGT
Molecular probe_CYP21A2	(FAM)-TCTGGAAAGCCCACAAGAAG-(RTQ1)
BGL	F:GTGCACCTGACTCCTGAGGAGA<br>R:CCTTGATACCAACCTGCCCAG
Molecular probe_BGL	(ROX)-AAGGTGAACGTGGATGAAGTTGGTGG-(BHQ-2)-3’

Supplementary Table S4. Primer sequences of the variants in *CFTR* gene

**Gene**	**Fragment amplified**	**Primer sequence (5′→3′)**
CFTR	CFTRdele2,3	F:GAGCTTCTGAAATTAATTGACCAC<br>R:GACCCATCATAGGATACAATG
CFTR (internal control)	10 exon	F:GACTTCACTTCTAATGA<br>R:GCCTGGCACCATTAAAGAA

Supplementary Table S5. The list of identified P/LP variants in the gene screening panel

Gene	Associated disorder	mRNA accession	Variant type	Nucleotide change	Protein change	dbSNP	No of cases	Allele frequency	gnomAD_ allele frequency v4.1.0	Northwest Russia, allele frequency	Ruseq allele frequency (healthy)	ACMG criteria
CYP21A2	Congenital adrenal hyperplasia	NM_000500.9	SNV	c.844G>T	p.Val282Leu	rs6471	5	0,0303	0,005	-	-	PS3, PM3_strong, PP2
CYP21A2	Congenital adrenal hyperplasia	NM_000500.9	SNV	c.710T>A	p.Val238Glu	rs12530380	1	0,00606	0,000002478	-	-	PS3, PM3_strong, PM2, PP2
CYP21A2	Congenital adrenal hyperplasia	NM_000500.9	SNV	c.955C>T	p.Gln319Ter	rs7755898	5	0,0303	0,0008961	0,00000	-	PVS1, PS3, PM3_strong, PM2, PP2
CYP21A2	Congenital adrenal hyperplasia	NM_000500.9	SNV	c.293-13C>G (legacy name I2splice)	-	rs6467	1	0,00606	-	-	-	PS3, PM3_strong, PM2, PP2
CYP21A2	Congenital adrenal hyperplasia	NM_000500.9	SV	NA	deletion/conversion of the gene		1	0,00606	-	-	-	well-established pathogenic variant
GJB2	Sensorineural nonsyndromic hearing loss	NM_004004.6	SNV	c.-23+1G>A	-	rs80338940	1	0,00606	0.0002804	-	-	PS3, PM3_strong, PP3
GJB2	Sensorineural nonsyndromic hearing loss	NM_004004.6	Indel	c.35del	p.Gly12fs	rs80338939	5	0,0303	0,00705	0,01837	0,01521	PVS1, PS4, PM3_strong + expert guidelines
GJB2	Sensorineural nonsyndromic hearing loss	NM_004004.6	SNV	c.101T>C	p.Met34Thr	rs35887622	2	0,01212	0,01204	0,01535	0,01823	PS3, PS4, PM3_strong, PP1_moderate, PP3 + expert guidelines
GJB2	Sensorineural nonsyndromic hearing loss	NM_004004.6	SNV	c.269T>C	p.Leu90Gln	rs80338945	1	0,00606	0,0006667	0,00219	0,001777	PS3, PM3_strong, PM2, PP1, PP3
SERPINA1	Antitrypsin deficiency	NM_000295.5	SNV	c.187C>T	p.Arg63Gly	rs28931570	2	0,01212	0,001785	-	-	PS3, PM3_strong, PM2 (unconventional name PiI)
SERPINA1	Antitrypsin deficiency	NM_000295.5	SNV	c.863A>T	p.Glu288Val	rs17580	4	0,02424	0,03636	0,00669	0,007113	well-established pathogenic variant (unconventional name PiS)

SERPINA1	Antitrypsin deficiency	NM_000295.5	SNV	c.1096G>A	p.Glu366Lys	rs28929474	3	0,01818	0,01586	-	-	well-established pathogenic variant (unconventional name PiZ)
ATP7B	Wilson-Konovalov syndrome	NM_000053.4	SNV	c.2605G>A	p.Gly707Arg	rs191312027	1	0,00606	0,00129	0,00000	0,0002962	PM3_strong, PM1, PM2, PP2, PP3
ATP7B	Wilson-Konovalov syndrome	NM_000053.4	Indel	c.2287_2288insCA	p.Phe763Ter	-	1	0,00606	-	-	-	PVS1, PM2
ATP7B	Wilson-Konovalov syndrome	NM_000053.4	SNV	c.3207C>A	p.His1069Gln	rs76151636	2	0,01212	0,0009435	0,00618	0,005651	PS3, PS4, PM3_strong, PM2, PP1_moderate, PP3
ATP7B	Wilson-Konovalov syndrome	NM_000053.4	SNV	c.3688A>G	p.Ile1230Val	rs200911496	1	0,00606	0,0006573	0,00000	0,0005931	PM3_strong, PM1, PM5, PP2, PP3
ATP7B	Wilson-Konovalov syndrome	NM_000053.4	SNV	c.4039G>A	p.Gly1347Ser	rs587783318	1	0,00606	0,00005394	-	-	PM1, PM2, PM3, PP2, PP3
CFTR	Cystic fibrosis	NM_000492.4	SNV	c.220C>T	p.Arg74Trp	rs115545701	1	0,00606	0,0009288	0,00044	0,0005942	VUS + ivacaftor efficacy expert guidelines
CFTR	Cystic fibrosis	NM_000492.4	SNV	c.254G>A	p.Gly85Glu	rs75961395	1	0,00606	0,00006338	-	-	PS3, PM3_strong, PM2, PP3 + expert guidelines
CFTR	Cystic fibrosis	NM_000492.4	SNV	c.1397C>G	p.Ser466Ter	rs121908805	1	0,00606	0,00000813	0,00000	0,0002973	PVS1, PM3_strong, PM2 + expert guidelines
CFTR	Cystic fibrosis	NM_000492.4	Indel	c.1521_1523del	p.Phe508del	rs113993960	1	0,00606	0,01193	0,00623	0,008021	PS3, PS4, PM3_strong, PM4, PP1 + expert guidelines
CFTR	Cystic fibrosis	NM_000492.4	Indel	c.2052dup	p.Gln685fs	rs121908746	1	0,00606	0,00003409	0,00000	0	PVS1, PM3_strong, PM2 + expert guidelines
ABCA4	Stargardt disease/retinitis pigmentosa	NM_000350.3	SNV	c.1957C>T	p.Arg653Cys	rs61749420	1	0,00606	0,00002666	0,00000	0	PS3, PM3_strong, PM1, PM2, PP1, PP3
ABCA4	Stargardt disease/retinitis pigmentosa	NM_000350.3	SNV	c.5693G>A	p.Arg1898His	rs1800552	1	0,00606	0,001764	0,00308	0,003264	PS3, PM3_strong, PM2
ABCA4	Stargardt disease/retinitis pigmentosa	NM_000350.3	SNV	c.5882G>A	p.Gly1961Glu	rs1800553	3	0,01818	0,003406	0,00746	0,009775	PS3, PM3_strong, PM1, PM2, PP3
SMN1	Spinal muscular atrophy		SV	deletion of the 7 ex			5	0,0303	-	-	-	well-established pathogenic variant

DHCR7	Smith-Lemli-Opitz syndrome	NM_001360.3	SNV	c.452G>A	p.Trp151Ter	rs11555217	3	0,01818	0,0007107	0,00658	0,005045	PVS1, PS3, PM3_strong, PM2
DHCR7	Smith-Lemli-Opitz syndrome	NM_001360.3	SNV	c.651C>A	p.Tyr217Ter	rs749076525	1	0,00606	0,00001053	0,00044	0,001186	PVS1, PM3_strong, PM2
GALT	Galactosemia	NM_000155.4	SNV	c.563A>G	p.Gln188Arg	rs75391579	2	0,01212	0,002541	0,00310	0,001781	PS3, PS4, PM3_strong, PM2, PP2, PP3
GALT	Galactosemia	NM_000155.4	SNV	c.974C>T	p.Pro325Leu	rs111033794	1	0,00606	0,000004957	-	-	PS3, PM3_strong, PM1, PM2, PP2, PP3
PKHD1	Polycystic kidney and/or liver disease, type 4	NM_138694.4	SNV	c.4199C>T	p.Ser1400Leu	rs191201723	1	0,00606	0,0000254	0,00000	0	PM1, PM2, PM3
PKHD1	Polycystic kidney and/or liver disease, type 4	NM_138694.4	SNV	c.7264T>G	p.Cys2422Gly	rs201881567	1	0,00606	0,0003489	0,00271	0,002083	PM3_strong, PM1, PM2, PP1_moderate, PP3
SLC26A4	Pendred syndrome	NM_000441.2	SNV	c.103C>T	p.Gln35Ter	rs1426427399	1	0,00606	-	0,00000	-	PVS1, PM2
SLC26A4	Pendred syndrome	NM_000441.2	SNV	c.7264T>G	p.Cys2422Gly	rs201881567	1	0,00606	0.0003489	0,00271	0,002083	PM1, PM2, PM5, PP3
PAH	Phenylketonuria	NM_000277.3	SNV	c.1222C>T	p.Arg408Trp	rs5030858	2	0,01212	0.001324	0,00980	0.006813	PS3, PM3_Strong, PM2, PP3
IDUA	Mucopolysaccharidosis I (Hunter syndrome) Hurler-Scheie	NM_000203.5	SNV	c.1205G>A	p.Trp402Ter	rs121965019	1	0,00606	0.001265	0,00050	0.0003113	PVS1, PS3, PM3_strong, PM2 + expert guidelines
IDUA	Mucopolysaccharidosis I (Hunter syndrome) Hurler-Scheie	NM_000203.5	SNV	c.208C>T	p.Gln70Ter	rs121965020	1	0,00606	0.0004906	0,00351	0.0008892	PVS1, PS3, PM3_strong, PM2 + expert guidelines
DMD	Duchenne-Becker muscular dystrophy		SV	c.(5325+1_5326-1)_(5586+1_5587-1)dup	-	-	1	0,00606				VUS: 1A (0 points), 2I (0,45 points), 3A (0 points)
ALPL	Hypophosphatasia	NM_000478.6	SNV	c.571G>A	p.Glu191Lys	rs121918007	1	0,00606	0.001413	0,00219	0.002370	PS3, PM3_strong, PM2, PP2, PP3
USH2A	Usher syndrome type 2A	NM_206933.4	SNV	c.2276G>T	p.Cys759Phe	rs80338902	1	0,00606	0.001460	0,00045	0.0002967	PS4, PM3_strong, PM2, PP1_moderate, PP3 + expert guidelines

ACADS	Short-chain acyl-CoA dehydrogenase deficiency	NM_000017.4	SNV	c.319C>T	p.Arg107Cys	rs61732144	1	0,00606	0.0005020	0,00044	0.0002974	PS4, PM3_strong, PM2, PP1_moderate, PP3 + expert guidelines
ACADM	Medium-chain acyl-CoA dehydrogenase deficiency	NM_000017.4	SNV	c.127G>A	p.Glu43Lys	rs147559466	1	0,00606	0.002414	0,00413	0.003111	PS3, PM3_strong, PM1, PM2, PP3
BTD	Biotinidase deficiency	NM_001370658.1	SNV	c.1552C>T	p.Arg518Cys	rs80338686	1	0,00606	0.0001604	-	-	PS3, PM3_strong, PM2, PP3
PLOD1	Ehlers-Danlos syndrome, kyphoscoliotic type		SNV	c.192G>A	p.Trp64Ter	-	1	0,00606	-	-	-	PVS1, PM2
SLC26A2	Diastrophic dysplasia/achondrogenesis type Ib		SNV	c.310del	p.Leu104Ter	-	1	0,00606	-	-	-	PVS1, PM2
